# A Noninvasive Menstrual Blood-Based Diagnostic Platform for Endometriosis Using Digital Droplet Enzyme-Linked Immunosorbent Assay and Single-Cell RNA Sequencing

**DOI:** 10.34133/research.0652

**Published:** 2025-04-01

**Authors:** Han Wang, Zhouyi Gan, Yueyue Wang, Dingmeng Hu, Lexiang Zhang, Fangfu Ye, Ping Duan

**Affiliations:** ^1^ Department of Obstetrics and Gynecology, The Second Affiliated Hospital and Yuying Children’s Hospital of Wenzhou Medical University, Wenzhou, Zhejiang 325027, China.; ^2^ Oncology Discipline Group, The Second Affiliated Hospital of Wenzhou Medical University, Wenzhou, Zhejiang 325027, China.; ^3^ Joint Centre of Translational Medicine, The First Affiliated Hospital of Wenzhou Medical University, Wenzhou 325035, China.; ^4^ Oujiang Laboratory (Zhejiang Lab for Regenerative Medicine, Vision, and Brain Health), Wenzhou Institute, University of Chinese Academy of Sciences, Wenzhou, Zhejiang 325000, China.; ^5^ Beijing National Laboratory for Condensed Matter Physics, Institute of Physics, Chinese Academy of Sciences, Beijing 100190, China.

## Abstract

Endometriosis is marked by the ectopic growth, spread, and invasion of endometrial tissue beyond the uterus, resulting in recurrent bleeding, pain, reproductive challenges, and the formation of nodules or masses. Despite advancements in detection methods like ultrasound and laparoscopy, these techniques remain limited by low specificity and invasiveness, underscoring the need for a highly specific, noninvasive in vitro diagnostic method. This study investigates the potential of using menstrual blood as a noninvasive diagnostic sample for endometriosis by targeting genetic and inflammatory markers associated with endometriosis lesions. A novel digital droplet enzyme-linked immunosorbent assay (ddELISA) was developed, leveraging SiO_2_ nanoparticles for the femtomolar-sensitive detection of inflammatory cytokines (OPN, IL-10, IL-6) in menstrual blood. Single-cell RNA sequencing revealed differentiation patterns across endometrial tissues and menstrual blood, affirming that menstrual blood replicates key inflammatory and immune properties of endometriosis. Furthermore, endometriosis menstrual blood endometrial cells derived from human menstrual blood displayed similar properties to endometrial stromal cells in endometriosis lesions, validating menstrual blood as a suitable in vitro diagnostic sample. In contrast to traditional ELISA, ddELISA supports multi-target detection with enhanced sensitivity and reduced processing time, allowing precise biomarker analysis from minimal sample volumes. Our ddELISA-based approach shows promise as a rapid, accessible, and accurate diagnostic tool for endometriosis, with potential for practical clinical application.

## Introduction

Endometriosis is characterized by the occurrence, expansion, and invasion of endometrial tissue beyond the uterus, resulting in recurrent bleeding, discomfort, reproductive challenges, and the formation of nodules or masses [1]. This chronic inflammatory disease markedly impacts women’s reproductive health and overall quality of life [2,3]. Despite advancements in detection design, such as ultrasound and laparoscopy, these methods remain limited by low specificity and high invasiveness [4,5]. Thus, there is an urgent need for a specific, noninvasive in vitro detection method. Recent advances in early cancer screening technologies, such as genetic testing, allow for initial screening through blood or urine samples, improving both accuracy and speed [6,7]. However, peripheral blood testing as a noninvasive auxiliary tool for endometriosis has shown low sensitivity and specificity, largely due to the nonsystemic nature of endometriosis and the lack of definitive clinical biomarkers. One prevailing explanation for endometriosis is the retrograde menstruation theory, which suggests that menstrual blood should be explored as a promising avenue for testing [8]. Therefore, developing a specialized, sensitive, and easily detectable platform for early screening and companion diagnosis of endometriosis remains crucial.

Single-cell RNA sequencing (scRNA-seq) has already mapped the cellular heterogeneous landscape of the human endometrium [9,10]. In endometrial epithelial cells from endometriosis lesions, ARID1A mutations have been documented to up-regulate pro-angiogenic factors, remodel endothelial junctions, and enrich lymphatic endothelial cells, further demonstrating the utility of scRNA-seq in understanding intercellular interactions [3,11,12]. Despite these advances, few studies have investigated the link between endometriosis and menstrual blood cell differentiation. As we continue exploring menstrual blood as a noninvasive diagnostic tool for endometriosis, we focus on determining whether it carries genetic markers associated with endometriosis lesions. Introducing new diagnostic technologies, such as biochips and polymerase chain reaction (PCR), has greatly facilitated faster, more precise disease diagnosis and treatment [13–15]. Digital enzyme-linked immunosorbent assay (dELISA), which isolates and identifies single protein molecules on substrates confined in microwells or droplets, has demonstrated sensitivity improvements of 100- to 1,000-fold over traditional ELISA methods [16–20]. Despite these advances, due to the influence of Poisson noise, the measurement results are not accurate at very low target concentrations [21–23]. Additionally, the need for specialized instruments and complex protocols limits their widespread adoption. Droplet microfluidics, designed to generate and manipulate numerous picoliter-scale discrete fluid reactions in immiscible phases [24–26], offers distinct advantages. Recent advances have made droplet-based dELISA (ddELISA) more accessible, with droplet analysis offering advantages like low reagent consumption and efficient loading [27]. They reduce reaction volumes to the picoliter scale, generating signals at locally high concentrations and enabling single-molecule counting. However, these assays still require buffer-switching workflows before emulsification, and the one-pot ddELISA strategy is subject to a double Poisson distribution due to the random dispersion of solid-phase carriers and target proteins, leading to many empty droplets [28–30]. Therefore, a practical ddELISA system for detecting inflammatory factors in menstrual blood that does not require specialized microarrays and complex workflows has yet to be developed.

This study was the first to demonstrate, through scRNA-seq analysis, that menstrual blood contained genetic markers associated with endometriosis lesions. Based on this finding, we developed a ddELISA system to detect the protein expression of inflammatory markers [osteopontin (OPN), interleukin-10 (IL-10), and IL-6] and their immunoregulatory roles in menstrual blood. The method employed fluorescein isothiocyanate (FITC)-labeled droplet microfluidics to construct amide-functionalized SiO_2_ nanoparticles (SiO_2_ NPs) and measure droplet encapsulation rates. Aqueous microcarriers, such as agarose microgels, were prepared using microfluidics, forming stable microbead complexes with the ELISA reaction solution. Preliminary in vitro OPN, IL-10, and IL-6 tests on clinical samples, including endometrial tissue from invasive laparoscopic procedures and noninvasively collected menstrual blood, demonstrated the assay’s feasibility. This was further supported by investigating the impact of macrophages in the immune microenvironment on inflammatory factors in endometriosis. Additionally, the detection limit (LOD) and sensitivity were as low as femtomolar, and the simultaneous detection of 3 targets was significantly better than traditional commercial ELISA. Notably, the concordance between results from noninvasively obtained menstrual blood samples and invasive endometrial tissue samples suggested that menstrual blood-derived endometrial tissue might replace surgical sampling, particularly with a representative protein screening panel of inflammatory markers, helping diagnose the inflammatory state of endometriosis in a noninvasive, specific, and sensitive manner.

## Results and Discussion

### Schematic representation of the digital immunoassay method utilizing SiO_2_ NPs for protein molecular detection via ddELISA

**Fig. 1.**
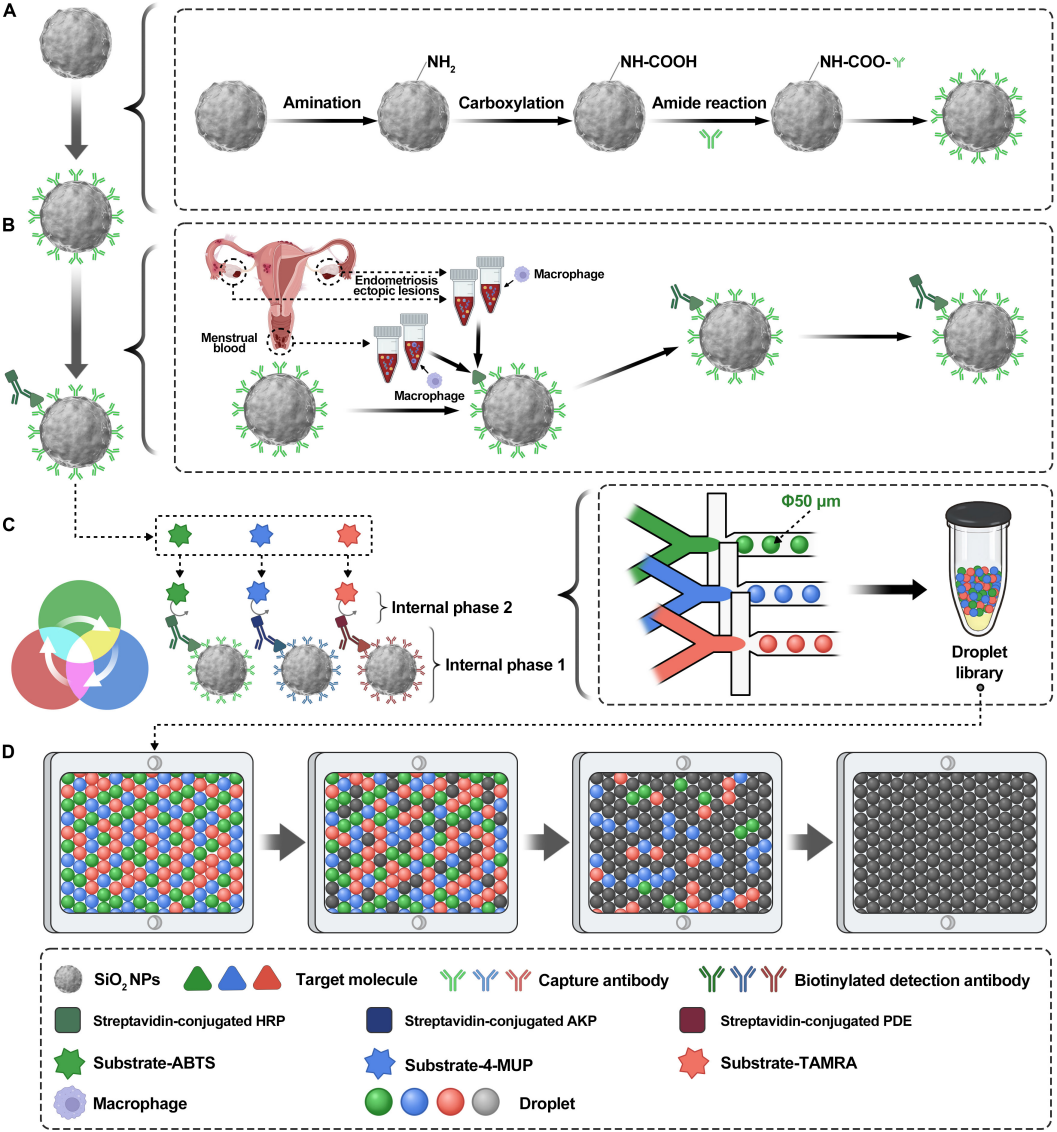
Schematic representation of the digital immunoassay method utilizing SiO_2_ NPs for protein molecular detection via ddELISA. (A and B) The technique involved encapsulating beads within monodispersed droplets of a specific size, which were then loaded into chambers to form a single-layer array for analysis. (C and D) This approach entails encapsulating beads within uniform droplets of precise dimensions, which are placed into small chambers to create monolayer arrays for analysis. The concentrations of 3 inflammatory factors (OPN, IL-10, and IL-6) were assessed in 4 primary endometrial cells. Furthermore, we explored the inflammatory interactions between primary endometrial cells and macrophages and the macrophage M2 polarization responses induced by primary endometrial cells.

Protein molecular detection via ddELISA typically involves a bead-based digital immunoassay approach. This method encapsulated beads within monodispersed droplets of specific sizes, which were then loaded into chambers to create a droplet array for analysis. Amidated SiO_2_ NPs coated with capture antibodies targeting molecules were introduced into biological samples, such as cell culture supernatant (Fig. 1A). Subsequently, biotinylated detection antibodies and streptavidin-conjugated enzymes [horseradish peroxidase (HRP), alkaline phosphatase (AKP), and phosphodiesterase (PDE)] were employed to label the target molecule, forming enzyme-labeled immune complexes (Fig. 1B). These complexes were resuspended in respective substrates (peroxidase substrate, ABTS; 4-methylumbelliferyl phosphate substrate, 4-MUP; phosphodiesterase PDE IV substrate, TAMRA). The resulting mixture was dispersed into droplets, ensuring that each droplet predominantly contained one bead due to the Poisson distribution, where a certain percentage of droplets contain multiple SiO_2_ NPs (Fig. 1C). Droplets generated from 3 channels of the droplet microfluidic chip were loaded into a single-layer chamber to form a fluorescence detection droplet array. Different fluorescent signals captured by 3 channels (HRP, AKP, and PDE) identify droplets positively for the target molecule. The sample content of the target molecule was then calculated based on the digitized signals of the droplets (Fig. 1D).

### The scRNA-seq analysis of endometriosis and menstrual blood

**Fig. 2.**
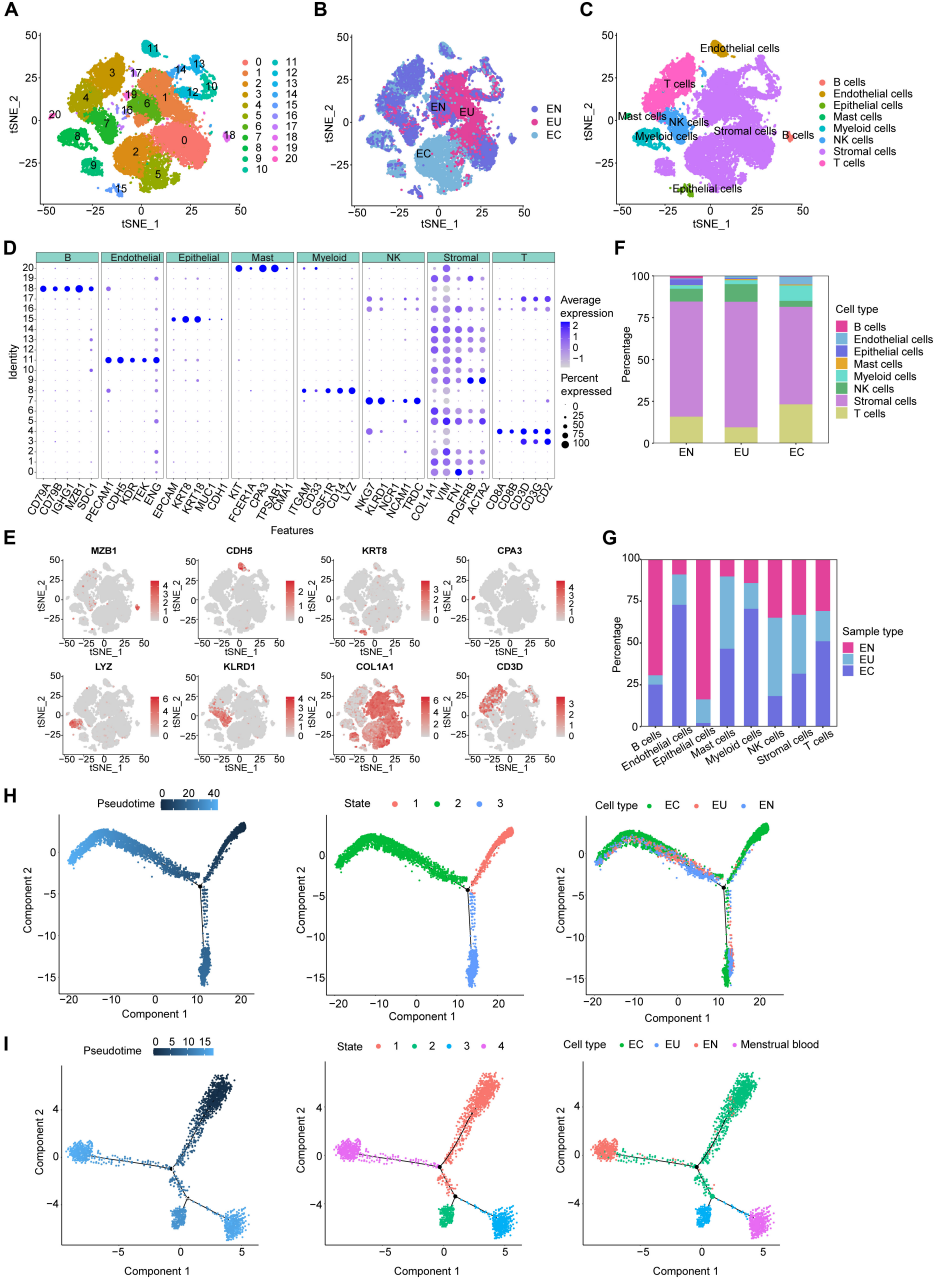
scRNA-seq analysis of endometriosis and menstrual blood. (A) The tSNE plots of 20 cellular clusters were identified in 28,085 cells. Each dot represented a cell. (B) tSNE plots of cells clustered by individual samples. (C) The tSNE plot identified 8 cell types in endometrial tissues. (D) Marker gene bubble diagram of marker genes of 8 cell subsets. (E) tSNE plot of 8 cell subsets marker genes. (F and G) The average proportion of assigned cell types in different groups was calculated. (H) Monocle 2 trajectory analysis of the ESCs from EN, EU, and EC samples, annotated pseudotime, cell state, and cell subgroups. (I) Monocle 2 trajectory analysis of the ESCs from EN, EU, EC, and menstrual blood samples, annotated pseudotime, cell state, and cell subgroups.

Current research on endometriosis primarily has focused on its pathogenesis. Although many genes have been identified as associated with the onset of endometriosis, there has been a lack of in-depth studies on diagnostic methods for the disease. Since these genes are difficult to detect in vitro, developing a noninvasive endometriosis detection method was particularly necessary. To elucidate changes in cell subpopulations associated with endometriosis, we performed scRNA-seq on endometrial tissues, including normal endometrial (EN), eutopic endometrium in endometriosis (EU), and ectopic endometrium in endometriosis (EC) tissues. After quality control and filtering (as detailed in the Methods section), we classified 28,085 cells into 20 clusters and visualized the data using *t*-distributed stochastic neighbor embedding (tSNE) (Fig. 2A). The dataset included 9,042 normal endometrial cells, 8,686 eutopic endometrial cells from endometriosis, and 10,357 ectopic endometrial cells from endometriosis patients. Significant interindividual heterogeneity was observed, with each sample forming distinct clusters (Fig. 2B). Based on cell type-specific marker genes, we identified 8 distinct cell populations (Fig. 2C): B cells (MZB1, CD79A, CD79B, IGHG1, SDC1), endothelial cells (CDH5, PECAM1, KDR, TEK, ENG), epithelial cells (KRT8, EPCAM, KRT18, MUC1, CDH1), mast cells (CPA3, KIT, FCER1A, TPSAB1, CMA1), myeloid cells (LYZ, ITGAM, CD33, CSF1R, CD14), natural killer (NK) cells (KLRD1, NKG7, NCR1, NCAM1, TRDC), stromal cells (COL1A1, VIM, FN1, PDGFRB, ACTA2), and T cells (CD3D, CD8A, CD8B, CD3G, CD2) (Fig. 2D and E and Fig. S1). The cell proportion data revealed the distribution of different cell types across samples (Fig. 2F and G). Stromal cells constituted the major subpopulation, with a significant decrease in stromal cells in EC compared to EN, and an increase in EU, underscoring their functional importance. In the extracellular matrix environment, epithelial cells were significantly reduced in both EU and EC compared to EN, while endothelial cells were significantly increased, suggesting a role for enhanced vasculature in endometriosis development. Regarding immune cells, B and NK cells significantly decreased in EC compared to EN, whereas myeloid cells, T cells, and mast cells significantly increased, indicating notable immune dysregulation in EC. Interestingly, the proportion of T cells was significantly lower in EU compared to EN, suggesting complexity in T cell functionality.

Unlike conventional sample grouping, we specifically analyzed EU samples to explore whether menstrual blood could reflect endometrial characteristics in vivo. The differentiation features of endometriosis cells had not yet been thoroughly studied in scRNA-seq data, so we focused on analyzing the differentiation patterns of stromal cells in EN, EU, and EC samples. To investigate this, we employed Monocle 2 for pseudotime trajectory analysis, categorizing the 3 groups based on the expression and transition characteristics of endometrial stromal cells (ESCs). As shown in Fig. 2H, stromal cells exhibited 3 distinct differentiation states during development. Surprisingly, we found that EC was the starting point of differentiation, progressing gradually toward EU and EN, which challenged our previous understanding that the EU was the starting point. Although we cannot determine whether the EU played a leading role before the onset of endometriosis, it became clear that after the onset of endometriosis, EU characteristics were altered in response. Surprisingly, EC appeared as the starting point of differentiation, gradually transitioning into EU, suggesting that post-endometriosis changes may influence gene expression in EU. To further validate the potential of menstrual blood as a noninvasive sample for endometriosis detection, we integrated menstrual blood data from dataset GSE203191 into our analysis and performed pseudotime trajectory analysis again (Fig. 2I). The results showed that EC remained the starting point of differentiation, progressively transitioning into EU. Notably, menstrual blood emerged as the terminal stage of EC differentiation and represented the next phase of EU differentiation, indicating that menstrual blood carries genetic markers from EU influenced by EC. In conclusion, through scRNA-seq analysis of endometriosis and menstrual blood, we confirmed the significant potential of menstrual blood as a noninvasive sample for endometriosis detection.

### E-MESCs replicated functions related to inflammation and macrophage activity observed in EESCs

**Fig. 3.**
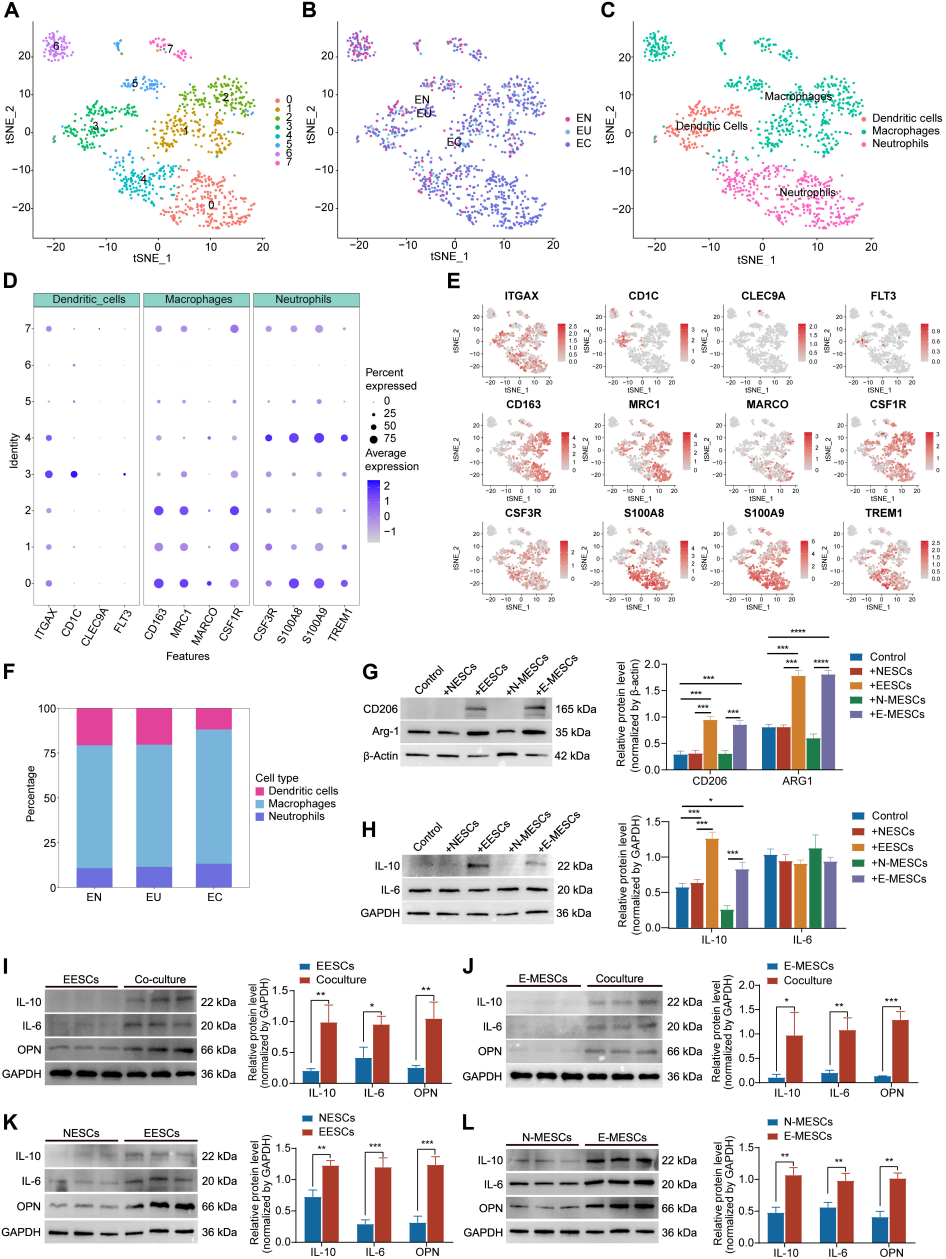
E-MESCs replicated the function of macrophages related to EESCs. (A) tSNE plot of the myeloid cell subset 1,168 cells, reclustered into 7 cell subpopulations. Each point represents a single cell. (B) tSNE plots of cells clustered by individual samples. (C) The tSNE plot identified 3 cell types in the myeloid cell subset. (D) Marker gene bubble diagram of marker genes of 3 cell subsets. (E) tSNE plot of 3 cell subset marker genes. (F) The average proportion of assigned cell types in different groups was calculated. (G) WB results show the protein expression of M2 polarization markers CD206 and ARG1 after macrophages were cocultured with NESCs, EESCs, N-MESCs, and E-MESCs. (H) WB results reveal changes in the inflammatory factors IL-10 and IL-6 following the coculture of macrophages with NESCs, EESCs, N-MESCs, and E-MESCs. (I) WB data indicate alterations in IL-10, IL-6, and OPN protein levels in EESCs after coculturing with macrophages. (J) WB analysis demonstrates IL-10, IL-6, and OPN expression shifts in E-MESCs post-coculture with macrophages. (K) WB shows IL-10, IL-6, and OPN protein variations between NESCs and EESCs. (L) WB highlights IL-10, IL-6, and OPN expression changes in N-MESCs and E-MESCs. All data are shown as the mean ± SD of 3 independent experiments (*P < 0.05, **P < 0.01, ***P < 0.001, ****P < 0.0001).

Given the significant alterations observed in myeloid cell subpopulations, we reclassified these cells for further analysis (Fig. 3A). Interindividual heterogeneity was apparent across tissue samples (Fig. 3B). We identified and characterized 3 major subgroups based on known myeloid lineage markers: dendritic cells, macrophages, and neutrophils (Fig. 3C). The expression of distinct marker genes for each subgroup was illustrated using bubble and tSNE plots (Fig. 3D and E): dendritic cells (ITGAX, CD1C, CLEC9, FLT3), macrophages (CD163, MRC1, MARCO, CSF1R), and neutrophils (CSF3R, S100A8, S100A9, TREM1). The relative proportions of these subgroups, as shown in Fig. 3F, revealed a significant increase in macrophage abundance in EC. This finding aligned with previous conclusions that extensive macrophage infiltration and activation in endometriosis triggered inflammatory responses. It was also demonstrated that endometriosis ectopic endometrial stromal cells (EESCs) promoted macrophage polarization toward the M2 phenotype, fostering malignant biological behaviors such as proliferation and metastasis [31].

Inhibiting macrophage activity can help decrease the production of pro-inflammatory cytokines, thereby reducing chronic inflammation associated with various diseases, including autoimmune disorders and chronic infections [32]. We further explored whether endometriosis menstrual blood endometrial cells (E-MESCs) had the same ability to replicate the functions of EESCs. Through Western blotting (WB) analysis of macrophage polarization after coculture, we found that coculture with either EESCs or E-MESCs significantly increased the expression of M2 markers CD206 and Arg1, indicating that E-MESCs could also promote macrophage polarization toward the M2 phenotype (Fig. 3G). Furthermore, coculture with EESCs and E-MESCs significantly elevated IL-10 levels in macrophages, while IL-6 levels remained unchanged, consistent with the cytokine profile of M2-polarized macrophages (Fig. 3H). WB analysis also revealed high expression of inflammatory factors IL-10, IL-6, and OPN in EESCs and E-MESCs after coculture with macrophages. Therefore, we concluded that E-MESCs retained cellular characteristics associated with EESCs regarding macrophage interaction (Fig. 3I and J). Prior sequencing data indicated that IL-10, IL-6, and OPN were significantly up-regulated in EESCs and E-MESCs compared to normal endometrial stromal cells (NESCs) and normal menstrual blood endometrial cells (N-MESCs) [33]. WB experiments further validated this conclusion (Fig. 3K and L). In the previous study, we discovered that OPN was significantly overexpressed in endometriosis and was implicated in its pathogenesis through the RhoA/reactive oxygen species (ROS) signaling pathway [33]. This finding suggested that OPN could serve as a promising target for the detection of endometriosis. Furthermore, we noted elevated levels of OPN in menstrual blood, which provided a solid theoretical foundation for proposing menstrual blood as a noninvasive in vitro sample for the detection of endometriosis in that study. Through our analysis of the immune microenvironment, consistent molecular characterizations in menstrual blood were associated with endometriosis lesions and promoted macrophage polarization and inflammatory responses. Notably, IL-10 and IL-6 were recognized as critical genes and inflammatory factors involved in macrophage polarization. Building on these findings, we proposed a detection array comprising OPN, IL-10, and IL-6 to develop a novel method for the in vitro detection of endometriosis.

### The ddELISA system construction for multi-component inflammatory factor detection

**Fig. 4.**
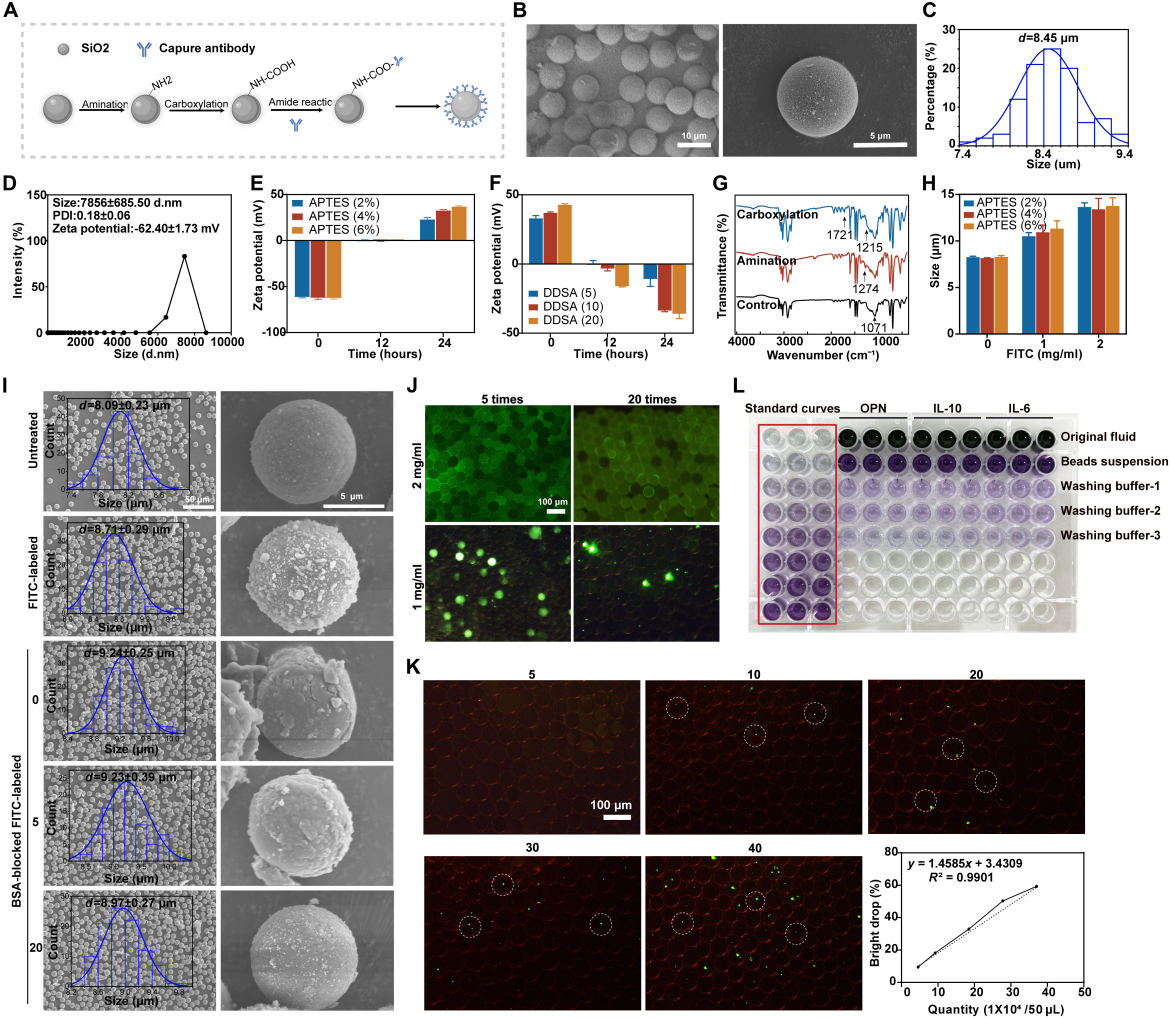
ddELISA system construction for multi-component inflammatory factor detection. (A) Synthesis route of the SiO_2_ NP binding to the capture antibody. (B) SEM images of SiO_2_ NPs. (C) Size distribution of SiO_2_ NPs observed in SEM images. (D) Size distribution and zeta potential progressions of SiO_2_ NPs, with PDI representing the polydispersity index. (E and F) Zeta potential progressions during modification by APTES and then DSSA. (G) FTIR spectra of SiO_2_ NPs, aminated SiO_2_ NPs, and carboxylated SiO_2_ NPs. (H) Size distribution of SiO_2_ NPs with different contents of FITC and APTES. (I) Overall and individual SEM images and particle size analysis of SiO_2_ NPs under different treatment conditions: untreated SiO_2_ NPs, FITC-labeled SiO_2_ NPs, BSA-blocked FITC-labeled SiO_2_ NPs, BSA-blocked FITC-labeled SiO_2_ NPs after 5 washes, and BSA-blocked FITC-labeled SiO_2_ NPs after 20 washes. (J) Fluorescence level of droplet background with varying FITC content and washing times. (K) Droplet encapsulation rate of FITC-labeled SiO_2_ NPs at different concentrations. Examples of luminous droplets are shown in the white circle. (L) The BCA assay was conducted to measure the protein concentrations in the original fluid, beads suspension, and washing buffers.

The ddELISA system was developed based on the traditional sandwich ELISA method, incorporating innovative nanoparticle molecules to replace the conventional large well-plate coating of capture antibodies. This approach offered several advantages, including reduced sample and antibody consumption, simplified washing steps, and shorter incubation times. As illustrated in Fig. 4A, SiO_2_ NPs were functionalized through amination and carboxylation, followed by antibody conjugation (targeting OPN, IL-10, and IL-6) via amide reactions. Scanning electron microscopy (SEM) images and particle size distribution analysis confirmed the monodispersity of SiO_2_ NPs, with an average diameter of approximately 8.45 μm (Fig. 4B and C). The size distribution showed a mean diameter of 7.85 ± 6.85 μm, and zeta potential measurements indicated a zeta potential of around −62.4 mV for the SiO_2_ NPs (Fig. 4D). The amination of SiO_2_ NPs was achieved using different concentrations of 3-aminopropyltriethoxysilane (APTES) in anhydrous ethanol (2%, 4%, 6%) over varying incubation times (12 and 24 h). Zeta potential results demonstrated successful modification after 24 h with both 4% and 6% APTES solutions, with no significant difference between the 2, identifying 24 h with 4% APTES as the optimal modification condition (Fig. 4E and Table S1). Carboxylation was carried out by incubating the SiO_2_ NPs in alkenylsuccinic anhydride (DSSA)/dimethyl sulfoxide (DMSO) solutions. Zeta potential measures indicated a proportional decrease in zeta potential with increasing DSSA concentrations and longer modification times, with the optimal conditions being 24 h and 20 mg/ml DSSA (Fig. 4F and Table S2). Additionally, size distribution analysis confirmed that neither APTES nor DSSA modifications significantly affected the size of the SiO_2_ NPs (Fig. S2A and B). Fourier transform infrared spectroscopy (FTIR) analysis further verified the successful modification of amine and carboxyl groups, with characteristic peaks for amines at 1,274 cm^−1^ and carboxyl groups at 1,215 and 1,721 cm^−1^ (Fig. 4G).

The dispersion of solid particles in the droplet array followed Poisson statistics, where droplets could either be empty or contain at most one template per droplet. To address the issue of SiO_2_ NP wastage due to distribution limitations, the aim was to optimize the particle number by labeling SiO_2_ NPs with FITC. The effect of APTES and FITC content on 1 × 10^9^ SiO_2_ NPs revealed that amination was a key factor. Zeta potential analysis showed that 4% and 6% APTES effectively modified the SiO_2_ NPs (Fig. S2C and Table S3). The size of SiO_2_ NPs influenced droplet stability. Nanoparticle size analysis indicated that the diameters were approximately 10 and 12 μm when FITC concentrations were 1 and 2 mg/ml, respectively (Fig. 4H). The observed increase in particle size may have been attributed to bovine serum albumin (BSA) blocking. SEM further examined particle size changes of dried SiO_2_ NPs under various conditions (Fig. 4I). The particle size of 1 mg/ml FITC-labeled SiO_2_ NPs increased to 8.71 ± 0.29 μm, roughly 0.6 μm larger than untreated particles. After BSA coating, the size increased to 9.24 ± 0.25 μm, a 1.15-μm increase from untreated particles and a 0.53-μm increase compared to non-BSA-treated FITC-labeled NPs. After 5 washes, the particle size was 9.23 ± 0.46 μm, and after 20 washes, it reduced to 8.97 ± 0.42 μm. Furthermore, FITC concentration (1 to 2 mg/ml) significantly impacted background fluorescence intensity in droplets. After 20 washes, 2 mg/ml FITC-labeled SiO_2_ NPs exhibited high background fluorescence, whereas 1 mg/ml FITC significantly reduced background fluorescence after the same washing cycle (Fig. 4J). One of the challenges for femtoliter-scale aqueous droplet platforms in ddELISA was the difficulty in encapsulating solid-phase carriers with diameters in the micrometer or sub-micrometer range, which could block microfluidic channels or sediment out of the droplets, interrupting the detection process. Our hydrogel droplet microfluidics addressed these challenges by providing robust chain support, thermal stability, biocompatibility, and mechanical strength for even distributing cargo. An agarose matrix containing reaction solutions was introduced into the aqueous phase to enhance droplet stability and improve NP dispersion. Stable droplet morphology was obtained when the agarose content ranged between 2% and 3% (Fig. S2D).

Given that ddELISA outperformed traditional large-volume ELISA formats in sensitivity, a simple polydimethylsiloxane (PDMS) microfluidic chip equipped with a flow-focusing device was designed to generate responsive agarose microcarriers. The detailed design of the microfluidic chip is illustrated in Fig. S2E. Under gentle heating conditions, this device split the agarose, SiO_2_ NPs, and substrate enzyme solution into droplets. The resulting droplets exhibited uniform shapes, with an average diameter of 50 μm and a corresponding volume of 65 pl (Fig. S2F). Although droplet size could be altered by adjusting microchannel dimensions and flow rates, previous studies showed no significant effect on responsiveness within the 50- to 150-μm range (Fig. S2G). Thus, a droplet size of 50 μm was selected, and a droplet array containing 7.6 × 10^5^ droplets from 50 μl of the reaction solution was generated to meet the LOD requirements. The effect of SiO_2_ NP concentration on positive droplet fluorescence intensity under various conditions was investigated, demonstrating a positive correlation between fluorescence intensity and the number of SiO_2_ NPs per unit volume (Fig. 4K). Poisson statistics indicated that at a SiO_2_ NP concentration of 4 × 10^5^, the theoretical distribution was 60%. To achieve over 90% luminescence efficiency, 6 × 10^5^ SiO_2_ NPs were selected for subsequent experiments. In the bicinchoninic acid (BCA) assay, we measured the protein concentrations in the original fluid, beads suspension, and washing buffers, respectively. The results indicated that most of the captured antibodies were bound to the SiO_2_ NPs rather than remaining in suspension. This finding underscored the effectiveness of SiO_2_ NPs in the antibody capture process (Fig. 4L). The aminated SiO_2_ NPs were conjugated with capture antibodies, and a standard curve from BCA protein assays showed a maximum capture efficiency of 42.2% (Fig. S2H). At this protein concentration on SiO_2_ NPs, only trace amounts of protein were detected in the washing solution, indicating that most capture antibodies were bound to the SiO_2_ NPs rather than remaining in suspension (Fig. S2I). The size and zeta potential of SiO_2_ NPs conjugated with capture and detection antibodies were not significantly affected by variations in antibody concentration, indicating the stability of SiO_2_ NPs after antibody conjugation (Fig. S2J and K). In conclusion, the functionalization of SiO_2_ NPs and the optimization of their usage in the ddELISA system were successfully achieved.

### Performance evaluation of the ddELISA system

**Fig. 5.**
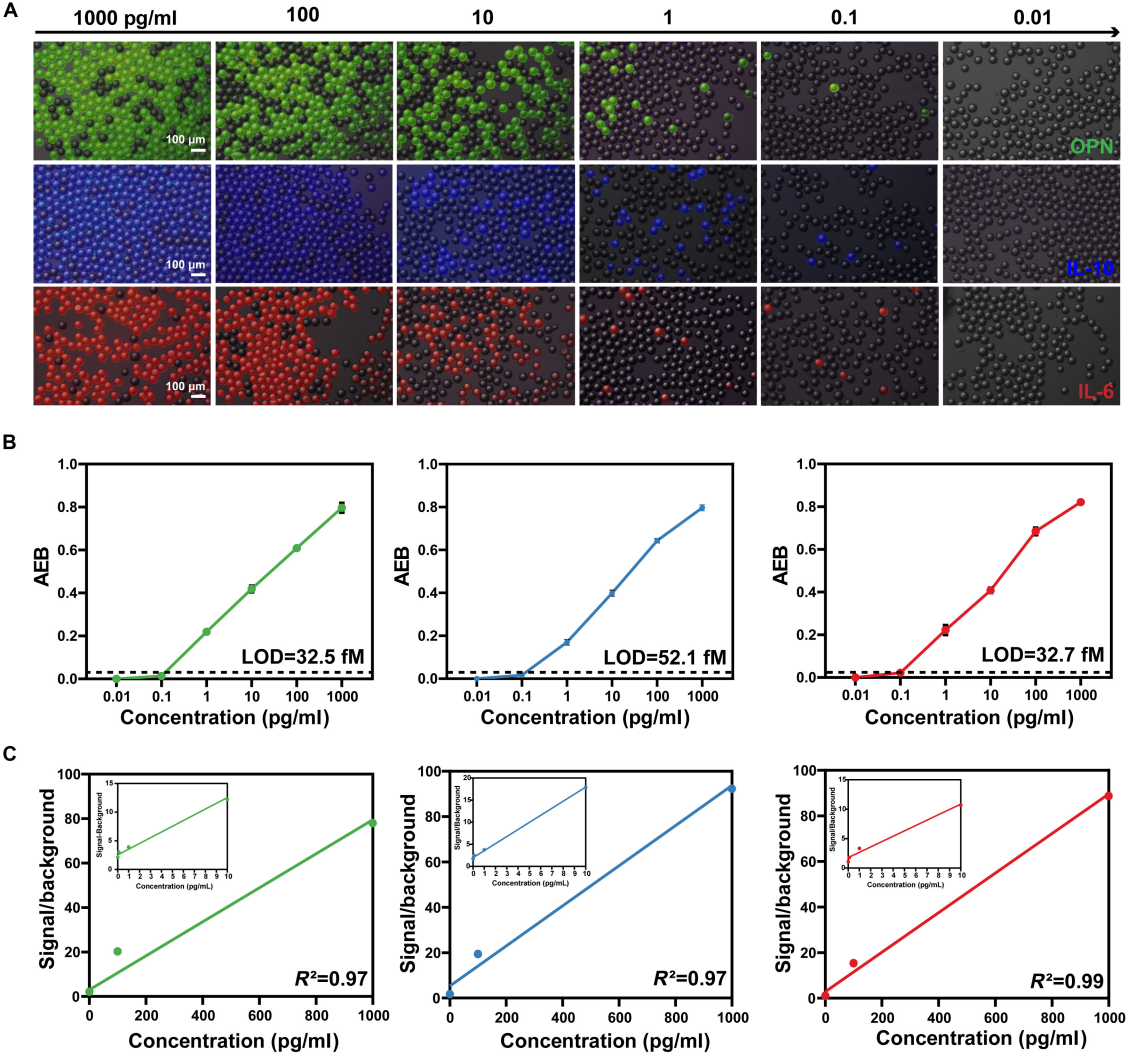
Performance evaluation of the ddELISA system. (A) Fluorescence images post-ddELISA under HRP-OPN (green), AKP-IL-10 (blue), and PDE-IL-6 (red) channels with target protein concentrations ranging from 1,000 to 0.01 pg/ml. (B) AEB corresponds to various target protein concentrations, where AEB increases with higher target protein concentrations. The LOD line indicates the mean ± SEM of 3 negative control samples without protein. (C) Background signal of the calibration curve: OPN, IL-10, and IL-6 amplified from 0.01 to 1,000 pg/ml (bottom), and OPN, IL-10, and IL-6 amplified from 0.01 to 10 pg/ml (top).

To assess the performance of ddELISA, we quantified the relative expression of 3 target proteins (OPN, IL-10, and IL-6) by measuring their levels and using standard curves. Each of the 3 inflammatory factors were labeled with a different fluorescent marker: HRP-OPN (green), AKP-IL-10 (blue), and PDE-IL-6 (red). Figure 5A displays fluorescent images of bead immunocomplexes encoded with varying concentrations of OPN, IL-10, and IL-6. As the protein concentrations decreased through dilution, the proportion of fluorescent droplets correspondingly declined. We correlated the AEB (average enzymes per bead) with the respective protein concentrations and plotted the standard curves for the inflammatory cytokines (Fig. 5B). At low protein concentrations (<0.1 pg/ml), most droplets did not contain target protein molecules. However, as the concentrations of target proteins increased (OPN > 0.48 pg/ml, IL-10 > 0.49 pg/ml, and IL-6 > 0.50 pg/ml), a minority of droplets began to contain multiple molecules. The LOD in the ddELISA method was determined by measuring blank samples without capturing antibodies, defined as 3 SDs above the background signal. The LOD for OPN, IL-10, and IL-6 was determined to be 32.5, 52.1, and 32.7 fM, respectively (Fig. 5B). Within the dynamic range, the signals for all 3 inflammatory factors demonstrated a strong linear relationship with the background, with *R*^2^ values of 0.97, 0.97, and 0.99, respectively (Fig. 5C).

### Evaluation of inflammatory factor alterations in endometrial and macrophages following coculture using the ddELISA

**Fig. 6.**
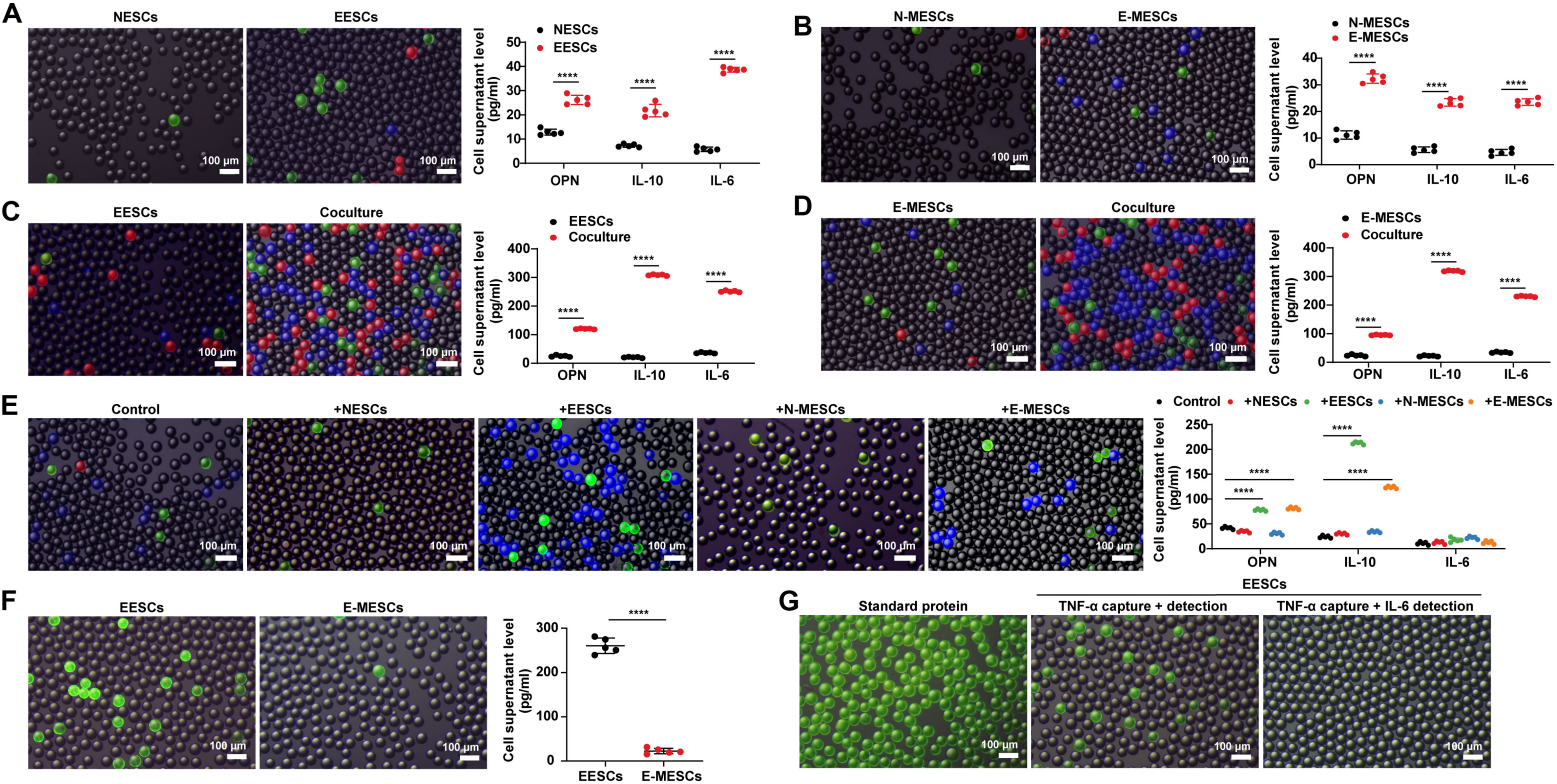
Evaluation of inflammatory factor using the ddELISA. (A) The ddELISA images in fluorescent channels and bright fields identify HRP-OPN-, AKP-IL-10-, and PDE-IL-6-tagged proteins and inflammatory factor measurements in NESC- and EESC-derived supernatants. (B) The ddELISA images identify HRP-OPN-, AKP-IL-10-, and PDE-IL-6-tagged proteins and inflammatory factor levels in N-MESCs and E-MESC supernatants. (C) The ddELISA images show HRP-OPN-, AKP-IL-10-, and PDE-IL-6-tagged proteins, along with inflammatory factor levels, in EESCs and EESCs cocultured with macrophage supernatants. (D) The ddELISA images show HRP-OPN-, AKP-IL-10-, and PDE-IL-6-tagged proteins, along with inflammatory factor levels, in E-MESCs cocultured with macrophage supernatants. (E) Detection of HRP-OPN-, AKP-IL-10-, and PDE-IL-6-tagged proteins and quantification of inflammatory factors in macrophage supernatants following coculture with NESCs, EESCs, N-MESCs, and E-MESCs. Post-ddELISA detection results are expressed by the merged images of HRP-OPN (green), AKP-IL-10 (blue), and PDE-IL-6 (red). (F) The ddELISA images in fluorescent channels and bright fields identify HRP-TNF-α-tagged proteins and inflammatory factor measurements in EESC- and E-MESC-derived supernatants. (G) The ddELISA images identified positive signals of HRP-TNF-α in both the TNF-α whole protein solution and the supernatant derived from EESCs. All data are shown as the mean ± SD of 3 independent experiments (NS is nonsignificant, **P < 0.01, ***P < 0.001, ****P < 0.0001).

To validate the practical application of the 3-color-coded assay, we measured inflammatory cytokines from 4 primary cell types (NESCs, N-MESCs, EESCs, and E-MESCs) for continuous cytokine detection. The results showed a significant increase in OPN, IL-10, and IL-6 concentrations in EESCs compared to NESCs, with values of approximately 26.1, 21.7, and 38.6 pg/ml, respectively, indicating a heightened inflammatory state in endometriosis (Fig. 6A). Similarly, OPN, IL-10, and IL-6 levels were significantly higher in E-MESCs than in N-MESCs, with concentrations of approximately 32.3, 23.4, and 23.5 pg/ml, respectively (Fig. 6B). Macrophages, as nonspecific immune cells, play a crucial role in the body’s response to pathogens, inflammation regulation, and tissue regeneration. Numerous studies have demonstrated that macrophages can trigger abnormal secretion of inflammatory factors [34–36]. We used ddELISA to measure the impact of coculturing endometrial cells from endometriosis with macrophages. In EESCs, coculturing led to a significant increase in inflammatory cytokine levels (Fig. 6C). This study was extended to E-MESCs, where we observed a similar elevation in inflammatory factors (increases ranging from 3.9- to 14.4-fold), comparable to the results in EESCs (increases ranging from 4.7- to 14.7-fold), indicating that macrophages can stimulate the secretion of inflammatory factors in endometriosis (Fig. 6D). To investigate the effect of endometriosis-derived endometrial cells on macrophage polarization, we cocultured macrophages with EESCs and E-MESCs and monitored cytokine changes using ddELISA (Fig. 6E). The results showed a significant increase in IL-10 levels in macrophages cocultured with EESCs or E-MESCs, with no significant change in IL-6 levels. NESCs and N-MESCs may lack inducers of macrophage secretion of inflammatory factors, while EESCs and E-MESCs induced M2-type polarization and secreted large amounts of IL-10. Additionally, we found that macrophages also stimulated increased secretion of OPN in both EESCs and E-MESCs. These findings indicated that E-MESCs exhibited similar functionality to EESCs, supporting using E-MESCs as a substitute for EESCs in assays.

To validate the feasibility of the ddELISA, we also measured the concentration of another key inflammatory factor, tumor necrosis factor-α (TNF-α), which had previously been found to be present at elevated levels in endometriosis [33]. The concentration of HRP-TNF-α in EESCs was 260.4 ± 17.4 pg/ml, while in E-MESCs it was 22.5 ± 6.2 pg/ml (Fig. 6F). Although TNF-α was present in endometriosis, the concentration of TNF-α in menstrual blood did not correlate with that in the lesions of endometriosis. This discrepancy indicated that menstrual blood could not effectively reflect the pathological characteristics of endometriosis using this marker. However, this result underscored the reliability of selecting OPN, IL-6, and IL-10 as detection markers in menstrual blood. Based on the linear requirements of the ddELISA for TNF-α detection, the specificity of the ddELISA assay was further evaluated. The HRP-TNF-α capture antibody was used to replace the HRP-IL-6 detection antibody in the experiment, and the negative results confirmed its specificity (Fig. 6G).

### Comparison between ddELISA and traditional ELISA

**Fig. 7.**
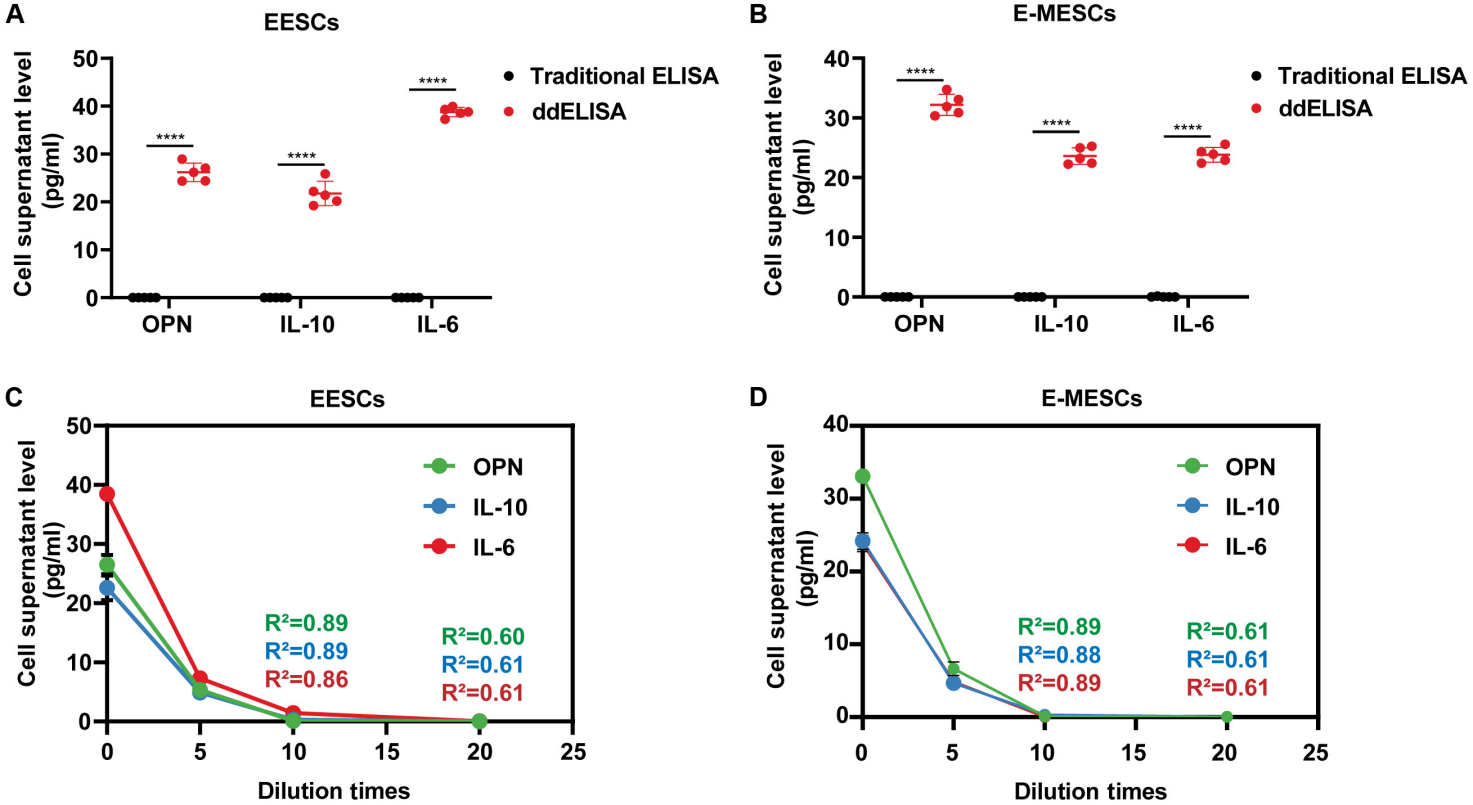
Comparison between ddELISA and traditional ELISA. (A and B) Comparison of traditional ELISA and ddELISA results for 100-fold diluted EESCs and E-MESCs samples. (C and D) Traditional ELISA concentration of EESCs and E-MESCs samples after gradient dilution with 0-, 5-, 10-, and 20-fold degrees. All data are shown as the mean ± SD of 3 independent experiments (****P < 0.0001).

By conducting a back-to-back comparison with traditional 96-well plate ELISA, we validated the feasibility and accuracy of ddELISA, with highly similar results between the 2 methods (Fig. S3). However, LOD was significantly different between the 2 approaches. ddELISA detected the original concentration of biological samples even when diluted 100-fold, whereas traditional ELISA almost failed to detect expression signals from EESCs and E-MESCs under the same conditions. In contrast, ddELISA maintained high sensitivity, yielding clear detection results (Fig. 7A and B). The sensitivity of traditional ELISA is limited when detecting low-concentration biological samples, with a linear range constrained to a dilution of less than 10-fold. Specifically, the concentrations of OPN, IL-10, and IL-6 in EESCs were 5.33, 4.88, and 7.30 pg/ml, respectively, and in E-MESCs, the concentrations were 6.65, 4.66, and 4.84 pg/ml, respectively. However, upon 10-fold dilution, the assay began to lose linearity, with concentrations of OPN, IL-10, and IL-6 in EESCs dropping to 0.09, 0.29, and 1.43 pg/ml (*R*^2^ = 0.89, 0.89, and 0.86), and in E-MESCs, the concentrations of these factors dropped to 0.11, 0.32, and 0.04 pg/ml (*R*^2^ = 0.60, 0.62, and 0.61). At 20-fold dilution, the traditional method lost linearity, producing anomalous results. The concentrations of OPN, IL-10, and IL-6 in EESCs were 0.04, 0.03, and 0.06 pg/ml (*R*^2^ = 0.89, 0.88, and 0.89), while in E-MESCs, the concentrations were 0.04, 0.04, and 0.05 pg/ml (*R*^2^ = 0.61, 0.61, and 0.61), only 0.01 pg/ml higher than background values (Fig. 7C and D). This limitation was likely due to the reduced capture efficiency of low-concentration molecular antigens by capturing antibodies in traditional ELISA. These results indicated that ddELISA retained accuracy in detecting biological samples and significantly improved sensitivity for limited sample quantities.

## Conclusion

This study demonstrates that menstrual blood can serve as a reliable noninvasive sample for diagnosing endometriosis by capturing the inflammatory and immune characteristics of endometrial lesions. Using a newly developed ddELISA system, we achieved highly sensitive detection of key inflammatory markers (OPN, IL-10, and IL-6) in menstrual blood at femtomolar levels, comparable to surgically collected samples. The ddELISA platform not only allows for simultaneous multi-target analysis but also simplifies the diagnostic process, reducing time and complexity while offering a cost-effective and accessible alternative. These findings highlight the potential of menstrual blood-based diagnostics as a transformative tool for endometriosis screening and monitoring, promising a patient-friendly, efficient approach for real-world applications. Future work will focus on optimizing the ddELISA system and further validating its clinical utility to improve accessibility and diagnostic precision for endometriosis.

## Methods

### Materials

Sodium hydroxide (NaOH), APTES, DMSO, morpholine ethanesulfonic acid (MES), FITC, and agarose were procured from Aladdin. DSSA, carbodiimide hydrochloride (EDC), and *N*-hydroxysuccinimide (NHS) were provided by Marklin. PDMS was obtained from Dow Chemical Company. BCA reagent was sourced from Beyotime. A 0.1% Tween solution, 5× phosphate-buffered saline (PBS) solution, Dulbecco’s modified Eagle’s medium (DMEM), and RPMI 1640 medium were purchased from Solarbio. Primary antibodies were obtained from Proteintech, including OPN (22952-1-AP), IL-10 (60269-1-Ig), IL-6 (21865-1-AP), GAPDH (10494-1-AP), and β-actin (81115-1-RR). ELISA kits were provided by MultiSciences for OPN (EK1135), IL-10 (EK110), and IL-6 (EK106). Capture antibodies were sourced from BioLegend, including OPN (505407), IL-10 (506802), IL-6 (501101), and TNF-α (570109). Biotin-labeled detection antibodies were also procured from BioLegend, including OPN (507501), IL-10 (501502), IL-6 (201202), and TNF-α (502901). Streptavidin-coupled enzymes, including HRP (P638970) and AKP (S639002), were obtained from Aladdin and Whatman, along with PDE (13604). Substrate enzymes were procured from MCE, including ABTS (HY-15902), 4-MUP (HY-D0994), and TAMRA (HY-151774).

### Characterizations

The morphology of SiO_2_ NPs was examined using SEM with a SU8010 instrument. Dimensional measurements were performed with a Malvern Zetasizer (ZEN3600). FTIR analysis was conducted using a Tensor II spectrometer. For droplet manipulation and observation, a combination of an Olympus microscope (BX53) equipped with an AcutYee fast camera (Optronics 1875-ST-143) and a Nikon A1 laser scanning confocal microscope was employed.

### Clinical specimens

This study strictly adhered to the ethical standards of the Second Affiliated Hospital of Wenzhou Medical University. It recruited 20 women diagnosed with endometriosis through laparoscopy and histological examination at the same institution. The presence of ectopic endometrial glands was confirmed through pathological examination of the patients. Tissue samples were obtained during surgery and promptly transferred to the laboratory. The control group consisted of 20 women without endometriosis or adenomyosis who were undergoing surgical treatment for benign diseases related to women’s health, such as uterine leiomyomas. The study includes 3 types of samples, each of normal endometrial tissues, eutopic endometrium in endometriosis tissues, and ectopic endometrium in endometriosis tissues for scRNA-seq analysis. Additionally, on the second day following the last menstrual period before surgery, menstrual blood was collected from each patient using a sterile menstrual cup, with the collection lasting for 2 h. A sample volume of 2 ml was required. After collection, the samples were preserved in sterile PBS solution on ice and were promptly transported to the laboratory for the extraction of endometrial cells. Detailed patient information is shown in Table S4. All tissue samples were obtained with the informed consent of the patients.

### Single-cell preparation

The samples (0.2 to 0.9 g) were added to gentleMACS c-tubes (Miltenyi Biotec) containing enzymatic base solution (100 μg ml^−1^ Liberase TH research grade, and 50 μg ml^−1^ deoxyribonuclease I, Hanks’ balanced salt solution, 10 mM Hepes, 30 mM taurine). Tissues are minced using scissors and automatically digested using gentleMACS Octo Dissociator (Miltenyi Biotec) with heaters. Single-cell suspensions of depleted endometrial cells were washed with a basal solution containing 20% fetal bovine serum (FBS; Gibco), filtered through a 70-μm nylon filter (BD Falcon), collected by centrifugation (330*g*, 10 min, 4 °C), and resuspended in a basal solution containing 0.2% FBS (Gibco). Each centrifugation was manually counted 3 times by Tepan Blue and resuspended at a concentration of ≥2 × 10^6^/ml. Single cells were processed using the Chromium Controller (10x Genomics) according to the manufacturer’s protocol.

### scRNA-seq process

Single cells were run on a 10x Chromium system (10x Genomics) followed by library preparation by LC Sciences, following the recommended protocol of the Chromium Single Cell 30 kit (v2 Chemistry). The libraries were run on a HiSeq4000 for Illumina sequencing. Postprocessing and quality control were performed using the 10x Cell Ranger software package (v1.2.0; 10x Genomics). Reads were aligned to the mm10 reference assembly (v1.2.0; 10x Genomics). Initial evaluation of EN using a 10x Cell Ranger reported 9,042 cellular barcodes with 2,918 median genes sequenced per cell at 56.4% sequencing saturation and an average of 47,126 reads per cell. Initial evaluation of EU using this software reported 8,686 cellular barcodes with a median of 3,107 genes sequenced per cell, a sequencing saturation of 61.3%, and an average of 42,116 reads per cell. Initial evaluation of EC using this software reported 10,357 cellular barcodes with a median of 2,031 genes sequenced per cell, a sequencing saturation of 62.8%, and an average of 38,145 reads per cell.

### Bioinformatic analysis of scRNA-seq data

Bioinformatics analysis of scRNA-seq was performed by LC-bio (Hangzhou, China). Briefly, gene expression matrices generated by the 10x Cell Ranger polymerization option were further analyzed using the R package Seurat (version 4.0) with default parameters. Data were filtered according to the following thresholds: uniquely expressed genes (nFeature_RNA) less than 200 or greater than 2,500, and percentage of mitochondrial genome content greater than 5%. The data were then normalized by conversion using a scale factor (default 10,000) and logarithmic conversion using the Seurat embedding function. Correlation analysis was performed using the RunPCA function of the Seurat package, and then the 3 datasets were combined. Cluster analysis was performed using the standard Seurat package program with a resolution of 1.2. The identified clusters were then visualized using the tSNE of the principal components in Seurat. The average gene expression matrix for each cluster was then retrieved, and differential expression between clusters was performed using the functionality implemented in FindAllMarkers to identify the top markers for each cluster at a high level (parameters: only. pos = FALSE, min. pct = 0.2, thresh. use = 0.2).

### Pseudotime trajectory analysis of scRNA-seq

Pseudotime trajectory was plotted using the default settings of the R package monocle, version 2.4. Pseudotime ordering was performed using the function “reduce dimension” with max_components set at 2 and reduction_method set as DDRTree. Next, the significantly affected genes were obtained from the top 50 markers among the clusters by using the function differentialGeneTest (fullModelFormulaStr = ~Pseudotime) and were plotted with the function plot_pseudotime_heatmap. The num_cluster was set at 4 to obtain 4 modules of significantly changed genes with similar trends according to their pseudotemporal expression patterns.

### Cell culture

Tissues underwent precise mincing and were enzymatically digested using 4% collagenase solution (type IV) at 37 °C for 45 to 60 min. Subsequently, the resultant mixture was filtered through stainless steel mesh sieves to isolate and purify cells. Following centrifugation at 1,200*g* for 3 min, the lower cell fraction was repeatedly washed with DMEM 3 times. Subsequently, the ESCs were placed in a DMEM complete medium and cultured at 37 °C with 5% CO_2_. For the isolation of menstrual blood endometrial cells, a Percoll density gradient centrifugation method was employed. Initially, the menstrual blood gathered was combined with PBS and then centrifuged at 1,500*g* for 5 min. The lower blood layer was washed 3 times with PBS. The purified blood was then resuspended in DMEM and slowly layered onto a centrifuge tube containing Percoll at an equal volume to DMEM. This was followed by centrifugation at 1,800*g* for 25 min. Then, the white membrane layer was aspirated and washed thrice with PBS. Finally, N-MESCs and E-MESCs were separately suspended in DMEM and cultured at 37 °C with 5% CO_2_. THP-1 cells were incubated in RPMI 1640 medium with regular passaging. Upon exposure to PMA (phorbol 12-myristate 13-acetate) for 72 h, the THP-1 monocytes effectively transformed into macrophages. These differentiated macrophages adhered to the culture dishes, altering cell morphology. Transwell inserts with a pore size of 0.4 μm, made of polycarbonate, were employed in the coculture setup. Within this arrangement, 4 types of primary endometrial cells were cocultured at an equal ratio with the differentiated macrophages.

### Western blotting

Whole-cell and nuclear proteins were isolated and transferred to a polyvinylidene fluoride membrane. This membrane underwent incubation with primary antibodies and secondary antibodies. Following this, the membrane was exposed to an enhanced chemiluminescence solution. The results obtained were then analyzed. The ratios of their gray values relative to GAPDH or β-actin, used as internal standards, were calculated to quantify the relative abundance of the target proteins.

### ELISA

Inflammatory factor levels in cell supernatants were quantified using an ELISA kit. Cultured target cells were harvested and centrifuged at 1,000*g* for 20 min to remove cellular debris. The concentrations of OPN, IL-10, and IL-6 in the supernatants were then assessed. All steps of the ELISA followed manufacturer procedures, including sample addition, incubation, washing, and color development. Absorbance at 450 nm was measured within 15 min. Inflammatory factor concentrations were calculated based on standard curves. Each condition was tested in triplicate, and the experiment was repeated 3 times.

### SiO_2_ NP modification

The SiO_2_ NPs were activated in a 0.1% NaOH solution for 20 min. APTES and anhydrous ethanol were chosen for the amination modification solution. SiO_2_ NPs (1 × 10^9^) were dissolved in a 4% APTES/anhydrous ethanol solution, shaken at 300 rpm for 24 h at room temperature (RT), and washed 3 times with anhydrous ethanol before amination modification. DSSA and DMSO were used for carboxylation. The aminated SiO_2_ NPs were dissolved in a 10 mg/ml dodecenylsuccinic anhydride (DDSA)/DMSO solution, shaken at 300 rpm for 24 h at RT, and then thoroughly rinsed thrice with DMSO before utilizing in the amide reaction. The amide reaction solution comprised MES, EDC, and NHS solubilized in ddH_2_O. The precise amounts of MES, EDC, and NHS in ddH_2_O were set at 9.76, 35, and 55 mg/ml. The mixture was shaken at 37 °C for 6 h at 300 rpm and followed by a triple rinse using a MES/Tween solution with a 0.05 M concentration. The morphology of SiO_2_ NPs was examined by SEM. Dimensional measurements were conducted utilizing a Nanopox Zetasizer, while FTIR analysis was carried out using a Tensor II spectrometer.

### FITC-labeled SiO_2_ NPs

FITC was dissolved in 4% APTES/anhydrous ethanol solution, maintaining an FITC concentration of 1 mg/ml, and shaken at 300 rpm for 24 h at RT, protected from light. After 24 h, 1 × 10^9^ SiO_2_ NPs activated by 0.1% NaOH solution were added and shaken at RT for 24 h under light protection. The anhydrous ethanol solution was washed 20 times to remove the unattached FITC molecules entirely. FITC-labeled SiO_2_ NPs were closed with a 3% BSA solution.

### Microfluidic chip fabrication

The microfluidic channels were designed using automated CAD software to ensure that the design included all necessary channels, chambers, and ports. The process involved fabricating silicon molds using photolithography. The silicon wafer was cleaned and coated with a uniform layer of photoresist. The designed mask was placed on the photoresist-coated silicon wafer and exposed to ultraviolet light to induce a chemical reaction. The exposed silicon wafer was developed to remove the unexposed photoresist and form the channel pattern. The channel pattern was etched onto the wafer using either dry or wet etching techniques to create the mold. This was followed by thoroughly mixing the PDMS substrate and curing agent in a 10:1 ratio and placing the mixture in a vacuum chamber to remove air bubbles. The degassed PDMS was poured into the fabricated silicon mold, covering all the channels. The PDMS was cured in an oven at 80 °C for 1 to 2 h until fully cured. Subsequently, the PDMS was carefully peeled from the mold, while the PDMS chip and glass slide were treated with oxygen plasma to improve surface activity. The treated PDMS chip and glass slide had to be aligned and pressed to strengthen the bond. The channels were inspected under a microscope to ensure that they were intact and free of blockages or defects. The flow properties and sealing of the microfluidic chip were then tested.

### ddELISA process

Amidated SiO_2_ NPs were utilized to couple with capture antibodies. The capture antibody solution, composed of capture antibody/ddH_2_O, underwent rotary shaking at 37 °C for 3 h. The suspension of SiO_2_ NPs was collected to test the efficiency of SiO_2_ NPs coupled with capture antibodies in BCA assays. Capture antibody concentration ranged from 0.5 to 2 mg/ml. A standard curve of capture antibody concentration versus OD_405_ absorbance values was plotted. Biological samples, including cell supernatants, were selected. Cells (1 × 10^6^) were sown per well and incubated for 48 h, after which the cell supernatants were collected. SiO_2_ NPs bound to capture antibodies were dissolved in 10 ml of 0.1% Tween/5× PBS, and 100 μl of cell supernatant was added. The mixture was rotated at RT for 2 h. Next, the solution was exposed to a biotin-labeled detection antibody solution and subjected to rotary shaking for 1 h at RT. Streptavidin-coupled luciferase was added at a specific concentration of 1 mg/ml and subjected to rotary shaking for 30 min at RT. The resulting solution represents internal phase solution 1, while the substrate enzyme corresponding to streptavidin-coupled luciferase constitutes internal phase solution 2. Agarose was heated to a liquid state at 37 °C and subsequently mixed with internal phase solutions 1 and 2 at concentrations of 0%, 1%, 2%, and 3%, respectively.

The internal and external phase solutions were drawn into glass syringes of appropriate specifications and connected to 3 peristaltic pumps. Polyethylene tubing was used to link the glass syringes with the oil/water (O/W) single-emulsion microfluidic chip. Flow rates for internal and external phases were set, and the peristaltic pumps were activated. The inward flow rate was maintained at 50 μl/h, while other parameters remained constant, and the outward flow rate was adjusted to 110 μl/h. As the inner and outer phase fluids converged in the microfluidic channel, viscous forces and interfacial tension caused the inner phase fluid to stretch and break, forming monodisperse emulsion droplets. Multiple channels were simultaneously utilized for multi-indicator testing to generate monodisperse emulsion droplets across different channels. AEBs were calculated to characterize enzyme activity on SiO_2_ NPs using the formula AEB = total enzyme activity/number of beads. Background signal calculation was usually employed to eliminate experimental noise and obtain accurate results. It involved measuring the fluorescence signal of the blank sample 3 times and averaging the value. Droplet manipulation and observation were achieved through a combination of an Olympus microscope equipped with an AcutYee fast camera and a laser scanning confocal microscope.

### Statistical analysis

All quantitative data were presented as averages accompanied by SDs (*n* = 3). Statistical significance was measured using Student’s *t* test, one-way analysis of variance (ANOVA), and 2-way ANOVA. In the graphical representations, differences were deemed significant at *P* < 0.05. NS is nonsignificant (*P* ≥ 0.05), *P < 0.05, **P < 0.01, ***P < 0.001, ****P < 0.0001.

## Data Availability

The data utilized in this study were derived from experiments conducted by our research group. All data essential for evaluating the conclusions of this study are available in the main text and supplementary materials. For any inquiries regarding data access, please contact the corresponding author.
