# Supplementary material for: A Noninvasive Menstrual Blood-Based Diagnostic Platform for Endometriosis Using Digital Droplet Enzyme-Linked Immunosorbent Assay and Single-Cell RNA Sequencing
Source: Research (Wash D C). 2025 Apr 1;8:0652. doi: 10.34133/research.0652 (PMC11961069; doi:10.34133/research.0652)
Supplement: Supplementary 1 — Figs. S1 to S3 Tables S1 to S4 [file research.0652.f1.docx]

**Supplementary Information:**

**A Non-Invasive Menstrual Blood-Based Diagnostic Platform for Endometriosis Using Digital Droplet ELISA and Single-Cell RNA Sequencing**

Han Wang, Zhouyi Gan, Yueyue Wang, Dingmeng Hu, Lexiang Zhang, Fangfu Ye, Ping Duan


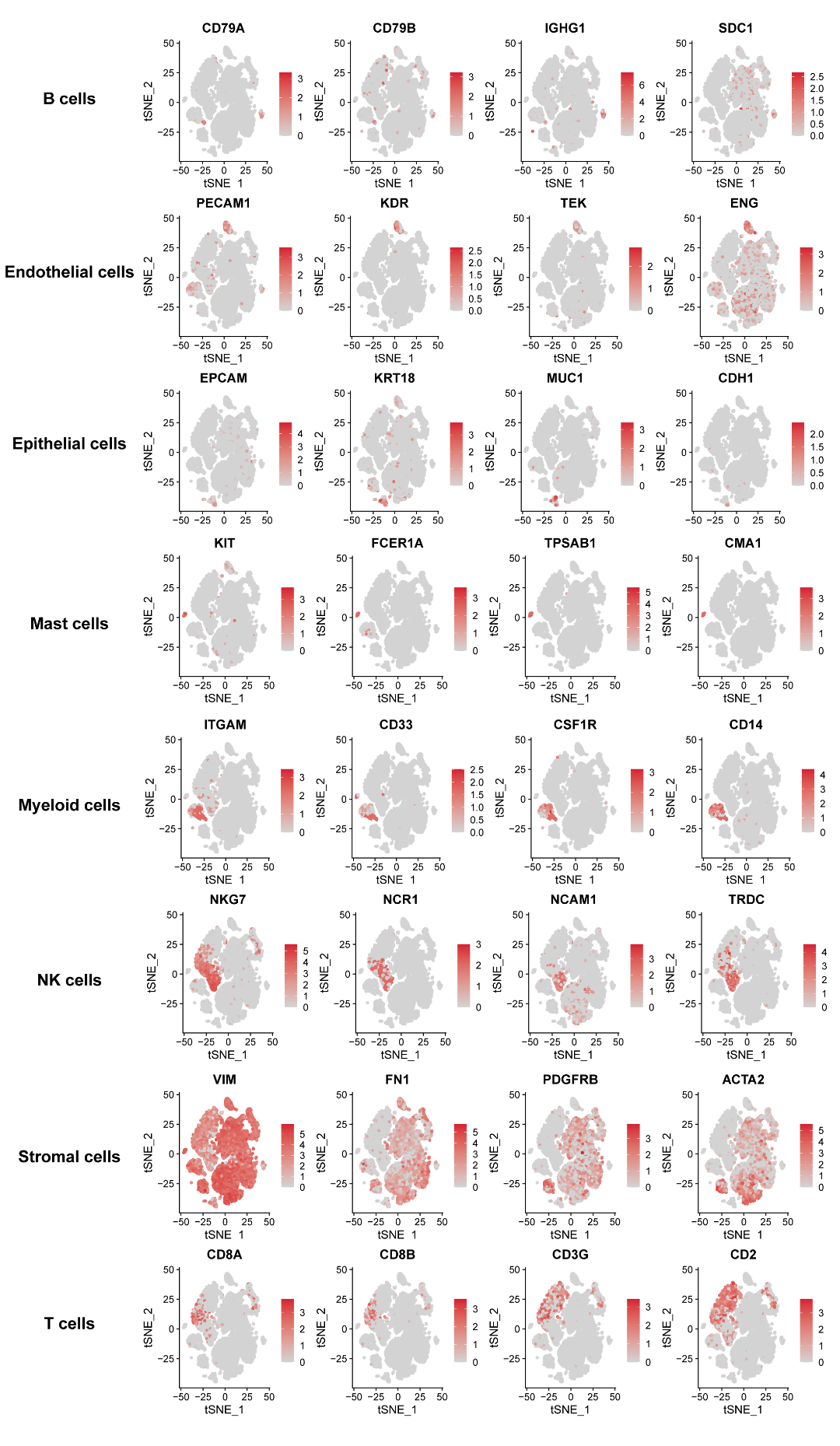


**Fig S1. The tSNE plot of 32 unique marker genes of UD.** The tSNE plot visualized the expression and distribution of 32 unique marker genes across the eight subpopulations of endometrial tissue.


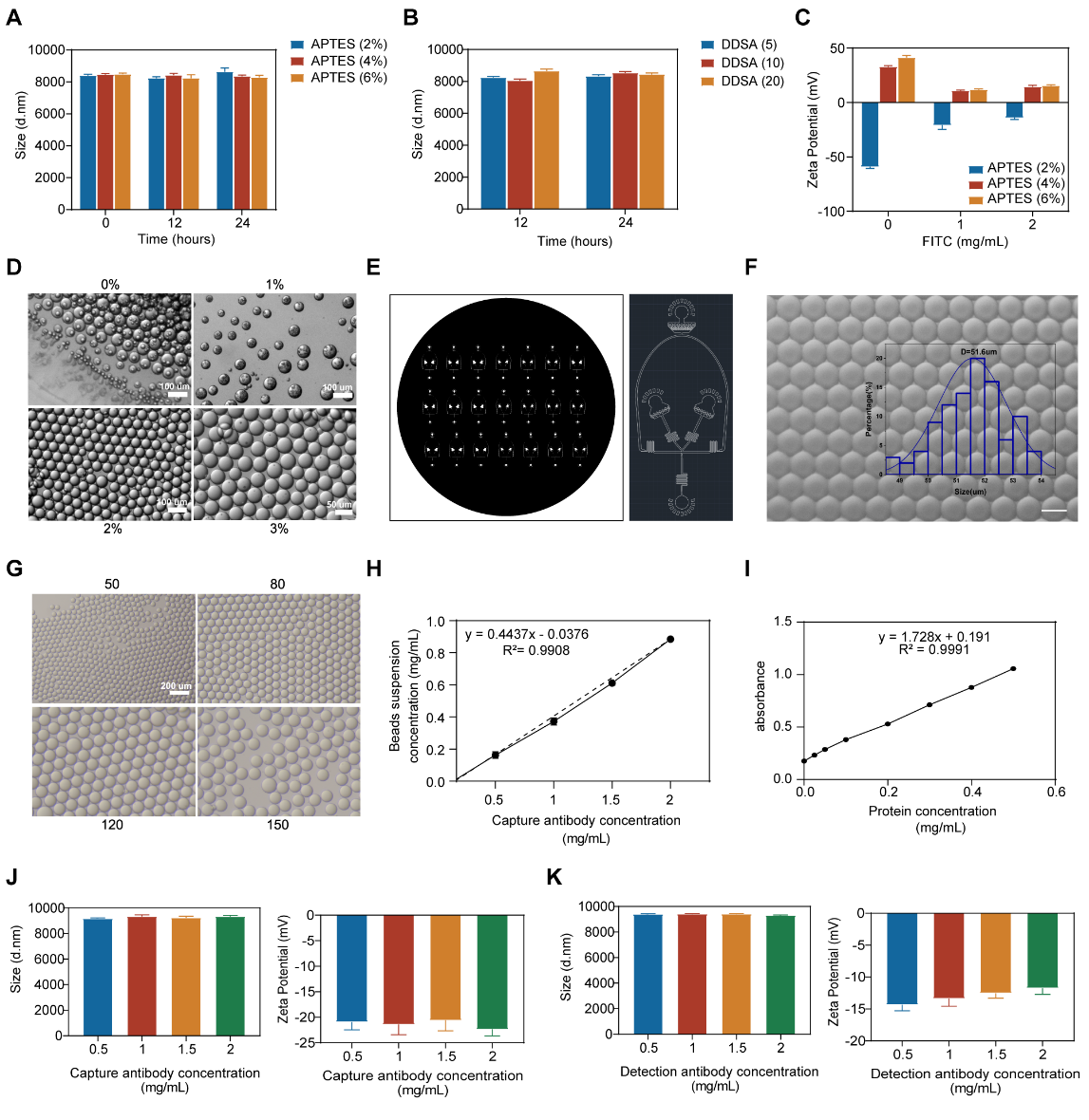


**Fig S2. Optimization of the ddELISA system.** (A) Particle size distribution analysis of SiO_2_ NPs after ammoniated modification at different times and APTES concentrations. (B) Size distribution analysis of carboxylated modified aminated-SiO_2_ NPs at different times and DDSA concentrations. (C) Zeta potential of aminated silica nanoparticles modified by varied concentrations of FITC labeling (0, 1, and 2 mg/mL) and APTES (2%, 4%, and 6%). (D) Effect of different agarose concentrations (0, 1%, 2%, and 3%) on the encapsulation of SiO_2_ NPs. (E) The overall image of the microfluidic chip diagram and details of a microfluidic chip unit. (F) Diameter measurement of droplet array. (G) Microfluidic devices generated droplets of different sizes (50, 80,120, and 150 μm). (H) Correlation of capture-antibody concentration with protein concentration in SiO_2_ NPs. (I) Standard curve of BCA protein concentration assay. Error bars, mean ± s.e.m. (n = 3). (J) Size distribution and Zeta potential analysis of SiO_2_ NPs conjugated with capture antibodies. (K) Size distribution and Zeta potential analysis of SiO_2_ NPs conjugated with detection antibodies.


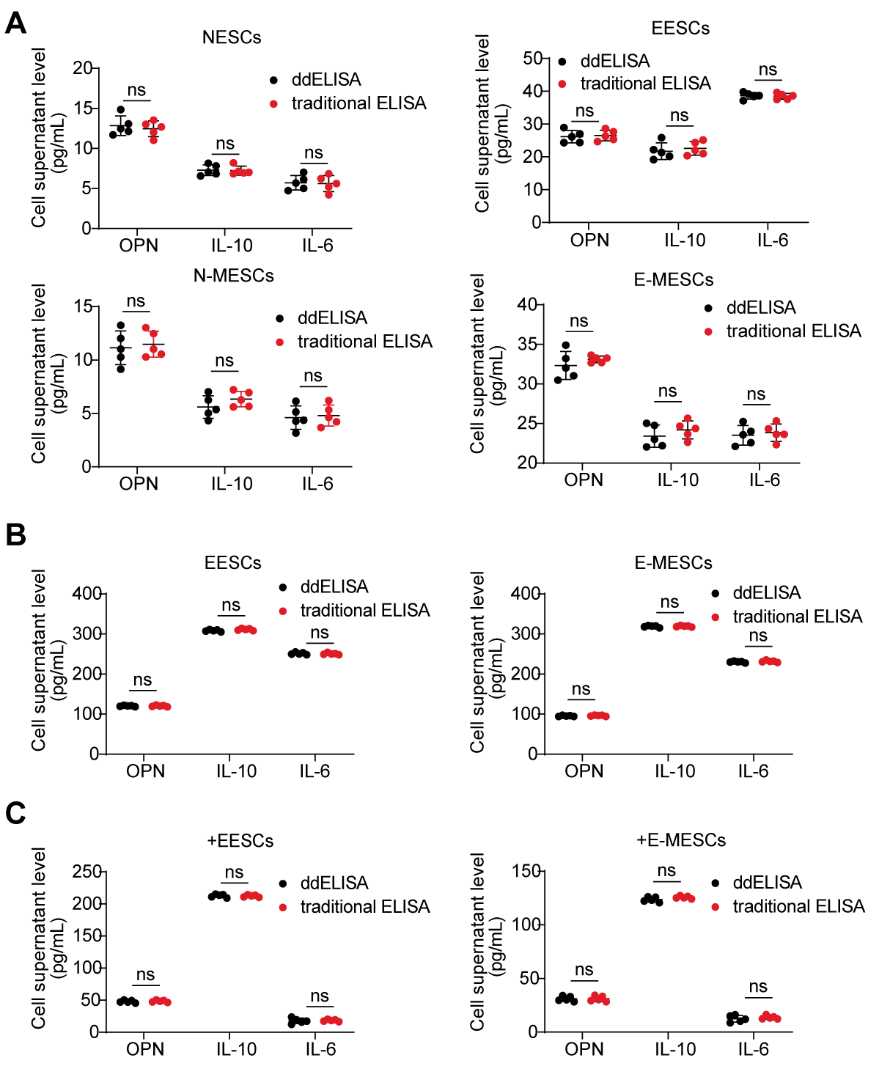


**Fig S3. Comparison of traditional ELISA results of undiluted samples and ddELISA results after 100-fold dilution.** (A) Comparison of traditional ELISA and ddELISA results of inflammatory factor levels in NESCs, EESCs, N-MESCs, and E-MESCs. (B) Inflammatory factor levels in EESCs and E-MESCs after co-culture with macrophages were compared using traditional ELISA and ddELISA results. (C) Traditional ELISA and ddELISA results were compared for the levels of inflammatory factors in macrophages co-cultured with EESCs and E-MESCs. All data are shown as the mean ± SD of three independent experiments (NS is nonsignificant).

**Table S1** Experimental amination modification details

| **TIME (h)** | **APTES (uL)** | **Size**  **(d.nm)** | **Zeta Potential (mV)** | **PDI** |
| --- | --- | --- | --- | --- |
| 12 | 100 | 8235.67±90.50 | +0.76±0.11 | 0.12±0.06 |
| 12 | 200 | 8422.33±104.10 | -0.19±0.43 | 0.18±0.05 |
| 12 | 300 | 8235.00±20885 | -0.08±0.09 | 0.13±0.09 |
| 24 | 100 | 8638.33±226.75 | +33.00±1.95 | 0.18±0.07 |
| 24 | 200 | 8350.67±82.68 | +36.93±0.68 | 0.10±0.05 |
| 24 | 300 | 8278.67±137.31 | +42.70±0.87 | 0.06±0.05 |

**Table S2** Details of the experimental carboxylation modifications

| **TIME**  **(h)** | **DDSA (mg)** | **Size**  **(d.nm)** | **Zeta Potential**  **(mV)** | **PDI** |
| --- | --- | --- | --- | --- |
| 12 | 5 | 8242.67±74.38 | +0.37±2.20 | 0.12±0.07 |
| 12 | 10 | 8056.67±103.47 | -3.39±1.59 | 0.21±0.05 |
| 12 | 20 | 8651.33±127.24 | +16.10±0.50 | 0.24±0.05 |
| 24 | 5 | 8329.33±86.09 | -10.87±5.38 | 0.20±0.05 |
| 24 | 10 | 8535.00±86.63 | -33.67±0.81 | 0.18±0.04 |
| 24 | 20 | 8442.33±84.13 | -42.70±2.48 | 0.20±0.03 |

**Table S3** Details of FITC-labeled SiO_2_ NPs in experiments

| **FITC (mg/mL)** | **APTES (uL)** | **Size**  **(d.nm)** | **Zeta Potential (mV)** | **PDI** |
| --- | --- | --- | --- | --- |
| 0 | 100 | 8246.67±102.34 | -59.01±1.63 | 0.21±0.04 |
| 0 | 200 | 9266.00±140.78 | +32.80±1.10 | 0.11±0.07 |
| 0 | 300 | 13635.00±458.13 | +41.23±1.91 | 0.24±0.06 |
| 1 | 100 | 8140.33±55.01 | -20.77±3.90 | 0.18±0.05 |
| 1 | 200 | 10934.67±767.40 | +10.74±0.81 | 0.23±0.05 |
| 1 | 300 | 13394.00±1172.01 | +11.70±0.95 | 0.11±0.05 |
| 2 | 100 | 8269.67±146.94 | -13.83±1.75 | 0.20±0.06 |
| 2 | 200 | 11315.67±±837.46 | +7.26±1.3 | 0.14±0.06 |
| 2 | 300 | 13755.33±893.16 | +10.57±0.96 | 0.18±0.06 |

**Table S4** Clinical characteristics of women with and without (control) endometriosis.

|  | **Control**  **(n=20)** | **Endometriosis**  **(n=20)** | ***P* value** |
| --- | --- | --- | --- |
| Age(years) | 38.05±3.44 | 37.20±3.70 | 0.456 |
| BMI(kg/m^2^) | 22.96±1.18 | 23.62±0.88 | 0.050 |
| Menstrual average cycle (days) | 29.05±2.28 | 29.70±1.59 | 0.302 |
| Menstrual duration (days) | 6.30±1.03 | 6.15±0.74 | 0.601 |
| ASRM stage |  |  |  |
| Stage 1 |  | 0/20 |  |
| Stage 2 |  | 3/20 |  |
| Stage 3 |  | 7/20 |  |
| Stage 4 |  | 10/20 |  |

Statistical analysis was performed using Student’s *t*-test. Data are mean±SD. ASRM, American Society for Reproductive Medicine.
